# The role of control region mitochondrial DNA mutations in cardiovascular disease: stroke and myocardial infarction

**DOI:** 10.1038/s41598-020-59631-x

**Published:** 2020-02-17

**Authors:** Miriam Umbria, Amanda Ramos, Maria Pilar Aluja, Cristina Santos

**Affiliations:** 1 Unitat d’Antropologia Biològica, Departament de Biologia Animal, Biologia Vegetal i Ecologia. Facultat de Biociències, Universitat Autònoma de Barcelona, Cerdanyola del Vallès, GREAB – Research Group in Biological Anthropology, Barcelona, Spain; 2 Faculdade de Ciências e Tecnologia, Universidade dos Açores (UAc), Ponta Delgada, Portugal; 3 Instituto de Investigação e Inovação em Saúde (I3S), Universidade do Porto, Porto, Portugal; 4 Instituto de Biologia Molecular e Celular (IBMC), Universidade do Porto, Porto, Portugal

**Keywords:** Genetic association study, Risk factors

## Abstract

Recent studies associated certain type of cardiovascular disease (CVD) with specific mitochondrial DNA (mtDNA) defects, mainly driven by the central role of mitochondria in cellular metabolism. Considering the importance of the control region (CR) on the regulation of the mtDNA gene expression, the aim of the present study was to investigate the role of mtDNA CR mutations in two CVDs: stroke and myocardial infarction (MI). MtDNA CR mutations (both fixed and in heteroplasmy) were analysed in two demographically-matched case-control samples, using 154 stroke cases, 211 MI cases and their corresponding control individuals. Significant differences were found, reporting mutations m.16145 G > A and m.16311 T > C as potential genetic risk factors for stroke (conditional logistic regression: p = 0.038 and p = 0.018, respectively), whereas the m.72 T > C, m.73 A > G and m.16356 T > C mutations could act as possible beneficial genetic factors for MI (conditional logistic regression: p = 0.001, p = 0.009 and p = 0.016, respectively). Furthermore, our findings also showed a high percentage of point heteroplasmy in MI controls (logistic regression: p = 0.046; OR = 0.209, 95% CI [0.045–0.972]). These results demonstrate the possible role of mtDNA mutations in the CR on the pathogenesis of stroke and MI, and show the importance of including this regulatory region in genetic association studies.

## Introduction

Cardiovascular disease (CVD) is one of the most widespread and common causes of death in the world. The onset and severity of these diseases are influenced by both genetic and environmental factors. Recent evidences associate mitochondrial dysfunction with several cardiovascular manifestations, mainly driven by the central role of mitochondria in cellular metabolism, particularly in energetically demanding tissues such as brain and heart^1,2^.

Human mitochondrial DNA (mtDNA) is 16.6-kb double-stranded circular DNA molecule that encodes for 13 electron transport chain (ETC) proteins, 2 ribosomal RNAs (rRNAs) and 22 transports RNAs (tRNAs). The control region (CR) encompasses the light and heavy strand promoters, the heavy strand origin of replication (O_H_), three conserved sequence blocks and the termination associated sequences (TAS)^3^. MtDNA is more susceptible than nuclear DNA to oxidative damage, probably due to the lack histone complex and an inefficient DNA repair mechanisms, which may serve as a protective barrier against external and internal noxious agents as reactive oxygen species (ROS)^4^. However, the hypothesis of direct damage by ROS is increasingly criticized and it is suggested that errors in mtDNA replication and repair may be the main cause of its high mutation rate (~10-fold greater than in nDNA)^5^.

Recent evidence have linked certain CVDs with specific mtDNA mutations including base substitutions^6–11^, deletions^12^, duplications^13^ and point or length heteroplasmy^14–17^ both in coding^6,9,10,12,14,15^ and noncoding region^6–9,11,13,16,17^ of mtDNA. In particular, mtDNA mutations located in CR have a potential importance since they may influence on the regulation of the mtDNA gene expression. In fact, several studies detected association of a great range of mtDNA variants (with negative or beneficial effect, both fixed or in heteroplasmy) and different diseases^6,9^.

In general, these disparities could occur because mtDNA mutations in the CR may not be directly tied to any form of pathology, but could be capable of influencing mitochondrial function through changes in the number of copies, inducing profound effects on the expression of mitochondrial-encoded gene transcripts and related enzymatic activities (complexes I, III, and IV)^18,19^.

The ability of mtDNA mutation to influence in the development of CVDs is directly related to its prevalence and the severity of its impact on mitochondrial function. In addition, several studies have demonstrated that due to the differences in the prevalence of the main etiological factors between intra- and extracranial arteries, the effect of mtDNA mutations in individuals with stroke or MI could be different^20–23^. The main aim of the present study was to investigate the role of CR mtDNA mutations (fixed or in heteroplasmy) in two CVDs; stroke and MI.

## Results

### Analysis of fixed and heteroplasmic mtDNA mutations with stroke

A detailed matrix of all mtDNA positions analyzed in stroke cases and controls are reported in Supplementary Table S1 and the frequencies of fixed mutations found are showed in Table 1. The percentages of m.16145 G > A and m.16311 T > C were overrepresented in stroke cases (5.2% and 18.2%, respectively) comparatively to controls (1.9% and 9.7%, respectively). After correction for the effect of CV risk factors with significant differences between stroke cases and controls (hypercholesterolemia^24^), significant association was still observed in m.16145 G > A (conditional logistic regression: p = 0.038; OR = 4.407, 95% CI [1.086–17.883]) and m.16311 T > C (conditional logistic regression: p = 0.018; OR = 2.417, 95% CI [1.165–5.016]), emerging as possible genetic risks factors for stroke (Table 1).

**Table 1:** Complete results of stroke fixed mtDNA mutation analysed.

	Position 16145 G > A	Position 16311 T > C
Controls: n = 154 (%)/Cases: n = 154 (%)	3 (1.9)/8 (5.2)	15 (9.7)/28 (18.2)
**Logistic Regression**^**a**^		
p-value	0.038	0.018
OR [95% CI]	4.407 [1.086–17.883]	2.417 [1.165–5.016]
**Stability analysis**		
CRS	G	T
Distribution in population database	G:97.2; A:2.8; GAP:0.02	T:76.9; C:23.0; Y:0.02; GAP:0.02
No. Hits phylogeny (PhyloTree.org)	37	137
No. Hits Soares *et al*.^50^	22	120
Probability of mutation	0,00205935	0,01123280
Nucleotide Conservation Index (%)	A:56.3; G:20.8; T:14.6; C:8.3	T:58.3; C:33.3; G:4.2; A:2.1; GAP:2.1

^a^Conditional regression model was performed considering significant covariates for stroke (hypercholesterolemia and mtDNA mutations).

**Figure 1:**
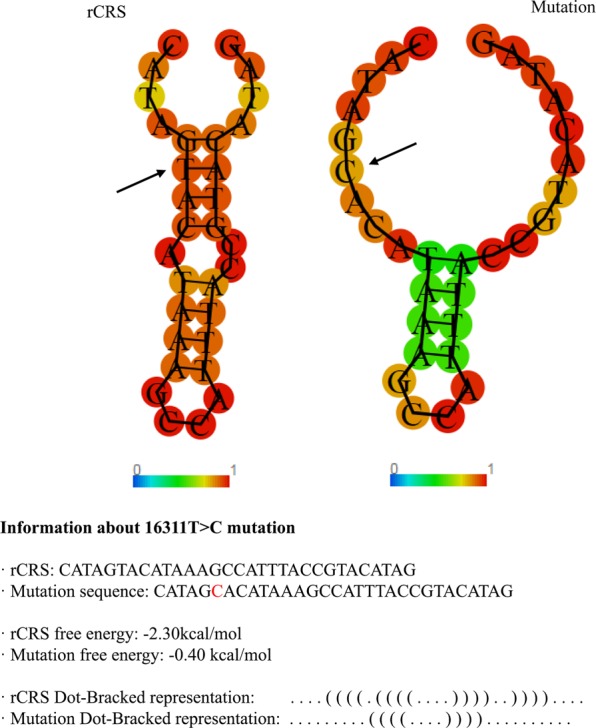
Revised Cambridge Reference Sequence (rCRS) *vs*. mutant structure and energy information. For m.16311 T > C, relevant secondary structure and energy information is listed along with a graphical drawing for both mutant and rCRS.

Stability analyses were performed to predict the impact of these mutations. Several measures as the number of hits in the mtDNA phylogeny, the probability of mutation, the frequency in the population database and the conservation index (CI) at nucleotide level, were calculated, and results are showed in Table 1. The results obtained revealed m.16145 G > A and m.16311 T > C as non-stable positions since they present a minimum of 37 hits in the phylogeny, a high probability of mutation, a high frequency of the variant in the population database (here denoted by minor allele frequency [MAF] > 5%) detected on m.16311 T > C or low-frequency (MAF 1–5%) in m.16145 G > A and a maximum nucleotide CI of 58% (Table 1). To infer about the impact of m.16145 G > A and m.16311 T > C on the stability of secondary structures of the mtDNA, a prediction of different structures with the Revised Cambridge Reference Sequence (rCRS) and mutant variant was performed. It seems that m.16311 T > C implies a conformational rearrangement, resulting in structure of Fig. 1 as the new predicted minimum free energy solution (−0.40 kcal/mol), causing a stability reduction of the region. No structural or thermodynamic differences were found for m.16145 G > A.

The distribution of the heteroplasmic positions between stroke cases and controls are reported in Table 2. Eighty-eight stroke cases (57.1%) and eighty-six controls (55.8%) presented point and/or length heteroplasmy (PH and LH, respectively), and no significant differences were obtained between groups. The most prevalent variant detected was a length heteroplasmy located in the poly-C tract of the HVRII (between positions 303–315 of the mtDNA), which was present in a 52% of stroke cases and in 46.7% of controls. Point heteroplasmies were found in six stroke cases and six controls, involving eight different positions of the mtDNA: 146, 150, 152, 185, 204, 16092, 16129 and 16399.

**Table 2:** Classification of the analyzed stroke and myocardial infarction (MI) individuals depending on the type(s) of heteroplasmy they present.

	Stroke Controls	Patients with stroke	p-value^a^	OR [95% CI]	MI Controls	Patients with MI	p-value^a^	OR [95% CI]
	n = 154 (%)	n = 154 (%)			n = 211 (%)	n = 211 (%)		
Homoplasmy	66 (42.86)	68 (44.16)	0.896	0.971 [0.621–1.517]	91 (43.13)	86 (40.76)	0.851	1.042 [0677–1.606].
Heteroplasmy	88 (57.14)	86 (55.84)	0.896	1.030 [0.659–1.610]	120 (56.87)	125 (59.24)	0.851	0.959 [0.623–1.478]
1PH	1 (0.65)	1 (0.65)	0.590	2.167 [0.130–36.121]	1 (0.47)	2 (0,95)	0.428	2.693 [0.232–31.217]
1LH	71 (46.10)	70 (45.45)	0.941	0.983 [0.627–1.542]	104 (49.29)	104 (49.29)	0.558	0.878 [0.569–1.356]
>1PH	0 (0.0)	0 (0.0)	—	—	0 (0.0)	0 (0.0)	—	—
>1LH	15 (9.74)	15 (9.74)	0.832	1.087 [0.502–2.356]	15 (7.11)	19 (9.00)	0.659	1.193 [0.545–2.614]
PH + LH	5 (3.25)	5 (3.25)	0.907	0.926 [0.257–3.337]	10 (4.74)	0 (0.0)	0.980	—
Total PH	6 (3.90)	6 (3.90)	0.914	1.067 [0.329–3.462]	11 (5.21)	2 (0.95)	0.022	0,046 [0.045–0.972]
Total LH	86 (55.84)	85 (55.19)	0.960	1.012 [0.645–1.588]	119 (56.40)	123 (58.29)	0.735	0,928 [0.604–1.427]

PH: point heteroplasmy; LH: length heteroplasmy.

^a^Logistic regression model was performed considering significant covariates for stroke (hypercholesterolemia and mtDNA heteroplasmy) and MI (hypertension, hypercholesterolemia and mtDNA heteroplasmy).

The analysis of stability performed to predict the impact of these heteroplasmic positions is presented in Table 3. In general, these mutations have a minimum of 16 hits in the phylogeny, were located in hotspots positions, have a high frequency of the minor variant in the population database and a low conservation index, indicating that these heteroplasmies have typical characteristics of non-stable positions. Morever, the frequency of the minor variant is relatively low (Table 3).

**Table 3:** Complete results of heteroplasmic positions analysed in stroke and myocardial infarction cases and controls samples (position, sample name, heteroplasmy type, heteroplasmy origin, distribution in population database, number of hits in mtDNA phylogeny [PhyloTree.org] and by Soares *et al*.^50^, probability of mutation and nucleotide Conservation Index).

Position	Sample name	Het	CRS	Mean proportion height peaks	Distribution in population database	No. Hits phylogeny (PhyloTree.org)	No. Hits Soares *et al*.^50^	Probability of mutation	Nucleotide Conservation Index (%)
146	Ca10_Stroke	T/c	T	83.33 T 16.67 C	T:81.4; C:18.4; A:0.1; GAP:0.1	121	109	0,01020313	C:41.7; T:31.3; A:27.1
150	Ca111_Stroke	T/c	C	68 T 32 C	C:88.2; T:11.7; G:0.1; GAP:0.1	74	63	0,00589722	C:43.8; G:27.1; A:18.8; T:10.4
152	Ca69_Stroke	C/t	T	52.94 C 47.06 T	T:70.4; C:29.5; GAP:0.1; G:0.02	196	157	0,01469625	C:47.9; T:35.4; A:16.7
185	Ca115_Stroke	G/a	G	66.67 G 33.33 A	G:94.6; A: 3.9; T: 1.1; C:0.3; GAP: 0.1; R:0.02	24	24	0,00224656	C:52.1; A:29.2; T:12.5; G:6.3
204	Ca79_Stroke	T/c	T	54.55 T 45.45 C	T:93.4; C:6.5; A:0.1; GAP:0.1; Y: 0,02	44	43	0,00402509	G:52.1; T:22.9; C:16.7; A:8.3
16129	Ca13_Stroke	G/a	A	73.33 G 26.67 A	G:84.6; A: 14.9; C: 0.4; R:0.02; GAP: 0.02	93	86	0,00805017	A:87.5; G:8.3; T:2.1; GAP:2.1
146	Co07_Stroke	T/c	T	69.23 T 30.77 C	T:81.4; C:18.4; A:0.1; GAP:0.1	121	109	0,01020313	C:41.7; T:31.3; A:27.1
146	Co68_Stroke	C/t	T	80 C 20 T	T:81.4; C:18.4; A:0.1; GAP:0.1	121	109	0,01020313	C:41.7; T:31.3; A:27.1
146	Co35_Stroke	T/c	T	66.67 T 33.33 C	T:81.4; C:18.4; A:0.1; GAP:0.2	121	109	0,01020313	C:41.7; T:31.3; A:27.2
152	Co16_Stroke	C/t	T	58.82 T 41.18 C	T:70.4; C:29.5; GAP:0.1; G:0.02	196	157	0,01469625	C:47.9; T:35.4; A:16.7
16092	Co40_Stroke	C/t	T	82.35 C 17.65 C	T:98.7; C:1.2; Y:0,1; GAP:0.02	16	17	0,00159131	A:41.7; T:33.3; C:20.8; GAP:4.2
16399	Co62_Stroke	G/a	A	83.33 G 16.67 A	A:97.4; G: 2.5; T: 0.02; C:0.02; GAP: 0.02; R:0.02	21	26	0,00243377	T:39.6; A:31.3; C:16.7; G;6.3; GAP:6.3
146	Ca120_MI	T/c	T	63.64 T 36.36 C	T:81.4; C:18.4; A:0.1; GAP:0.1	121	109	0,01020313	C:41.7; T:31.3; A:27.1
152	Ca100_MI	C/t	T	52.94 C 47.06 T	T:70.4; C:29.5; GAP:0.1; G:0.02	196	157	0,01469625	C:47.9; T:35.4; A:16.7
73	Co6_MI	G/a	A	76 G 24 A	G:80.8; A:19.1; GAP:0.1; C:0.02	12	11	0,00102967	A:41.7; C:35.4; G:22.9
146	Co56_MI	T/c	T	66.67 T 33.33 C	T:81.4; C:18.4; A:0.1; GAP:0.1	121	109	0,01020313	C:41.7; T:31.3; A:27.1
150	Co17_MI	T/c	C	86.20 T 13.80 C	C:88.2; T:11.7; G:0.1; GAP:0.1	74	63	0,00589722	C:43.8; G:27.1; A:18.8; T:10.4
150	Co123_MI	T/c	C	82.76 T 17.24 C	C:88.2; T:11.7; G:0.1; GAP:0.1	74	63	0,00589722	C:43.8; G:27.1; A:18.8; T:10.4
152	Co27_MI	T/c	T	58.82 T 41.18 C	T:70.4; C:29.5; GAP:0.1; G:0.02	196	157	0,01469625	C:47.9; T:35.4; A:16.7
152	Co184_MI	T/c	T	52.38 T 47.62 C	T:70.4; C:29.5; GAP:0.1; G:0.02	196	157	0,01469625	C:47.9; T:35.4; A:16.7
204	Co156_MI	T/c	T	83.33 T 16.67 C	T:93.4; C:6.5; A:0.1; GAP:0.1; Y: 0,02	44	43	0,00402509	G:52.1; T:22.9; C:16.7; A:8.3
16092	Co62_MI	C/t	T	82.35 C 17.65 C	T:98.7; C:1.2; Y:0,1; GAP:0.02	16	17	0,00159131	A:41.7; T:33.3; C:20.8; GAP:4.2
16093	Co106_MI	C/t	T	72.22 C 27.78 T	T:93.5; C:6.4; Y:0,1; GAP:0.02	55	79	0,00739493	T:64.6; A:20.8; G:6.3; GAP:4.2; C:4.2
16129	Co129_MI	G/a	A	81.81 G 18.19 A	G:84.6; A: 14.9; C: 0.4; R:0.02; GAP: 0.02	93	86	0,00805017	A:87.5; G:8.3; T:2.1; GAP:2.1
16399	Co94_MI	G/a	A	83.33 G 16.67 A	A:97.4; G: 2.5; T: 0.02; C:0.02; GAP: 0.02; R:0.02	21	26	0,00243377	T:39.6; A:31.3; C:16.7; G;6.3; GAP:6.3

### Analysis of fixed and heteroplasmic mtDNA mutations with MI

MtDNA positions studied for MI cases and controls are available in Supplementary Table S1 and frequencies of fixed mutations are reported in Table 4. The m.72 T > C, m.73 A > G and m.16356 T > C were more frequent in MI controls (12.3%, 49.3% and 3.3%, respectively) than cases (7.6%, 38.9% and 1.4%, respectively). When corrected for the effect of CV risk factors with significant differences between MI cases and controls (hypertension and hypercholesterolemia^24^), significant association was observed in these tree mutations (m.72 T > C: conditional logistic regression: p = 0.001; OR = 0.041, 95% CI [0.006–0.290], m.73 A > G: conditional logistic regression: p = 0.009; OR = 0.009, 95% CI [0.307–0.843] and m.16356 T > C: conditional logistic regression: p = 0.016; OR = 0.091, 95% CI [0.013–0.639]), emerging as possible protective genetic factors for MI (Table 3).

**Table 4:** Complete results of fixed mtDNA mutations in myocardial infarction cases and controls analysed.

	Position 72 T > C	Position 73 A > G	Position 16356 T > C
Controls: n = 211 (%)/Cases: n = 211 (%)	26 (12.3)/16 (7.6)	104 (49.3)/82 (38.9)	7 (3.32)/3 (1.42)
**Logistic Regression**^**a**^			
p-value	0.001	0.009	0.016
OR [95% CI]	0.041 [0.006–0.290]	0.509 [0.307–0.843]	0.091 [0.013–0.639]
**Stability analysis**			
CRS	T	A	T
Distribution in population database	T:97.4; C:2.4; G:0.1; GAP:0.1	G:80.8; A:19.1; GAP:0.1; C:0.02	T:97.5; C:2.5; GAP:0.02
No. Hits phylogeny (PhyloTree.org)	9	12	15
No. Hits Soares *et al*.^50^	6	11	19
Probability of mutation	0,00056164	0,00102967	0,00177853
Nucleotide Conservation Index (%)	T:77.1; GAP:8.3; A:6.3; C:4.2; G:4.2	A:41.7; C:35.4; G:22.9	T:79.2; C:12.5; A:6.3; GAP:2.1

^a^Conditional regression model was performed considering significant covariates for MI (hypertension, hypercholesterolemia and mtDNA mutations).

In order to predict the impact of these mutations, several measures were calculated to analyze the stability of each position, and results are showed in Table 4. The results obtained revealed that m.72 T > C, m.73 A > G and m.16356 T > C are non-stable positions since they present a minimum of 9 hits in the phylogeny, a high probability of mutation, a high frequency of the minor variant in the population database (MAF > 5%) for m.73 A > G and low-frequency (MAF 1–5%) for m.72 T > C and m.16356 T > C, and a maximum nucleotide CI of 79% (Table 4). Using the proposed previously method to predict the impact of these three mutations on the stability of secondary structure of the mtDNA, it seems that m.72 T > C, m.73 A > G and m.16356 T > C led to a folded structure with the same minimum free energy as the rCRS structure, which means that these mutations do not condition the stability of the region.

Classification of heteroplasmic positions in MI cases and controls is available in Table 2. One hundred twenty-five MI cases (59.2%) and one hundred and twenty controls (56.8%) presented point and/or length heteroplasmy, being the length heteroplasmy located in the poly-C tract of the HVRII the most prevalent variant in both MI cases (54.03%) and controls (48.34%). In this analysis, point heteroplasmy was significantly more frequent in MI controls (n = 11; 5.21%) than cases (n = 2; 0.94%) (logistic regression: p = 0.046; OR = 0.209, 95% CI [0.045–0.972]) even correcting for the effect of MI risk factors (hypertension and hypercholesterolemia^24^). These heteroplasmic positions involve nine different positions of the mtDNA: 73, 146, 150, 152, 204, 16092, 16093, 16129 and 16399.

The stability analysis to identify the impact of these point heteroplasmic positions is presented in Table 3. All of them were considered non-stable positions. As previously stated, these positions presented a minimum of 12 hits in the phylogeny, were located in hotspots positions, have a high frequency of the minor variant in the population database and low conservation index at nucleotide level. No different trends were observed between stability of these positions in MI cases and controls. Morever, the frequency of the minor variant is relatively low (Table 3).

### Distribution of mtDNA mutations between haplogroups

**Figure 2:**
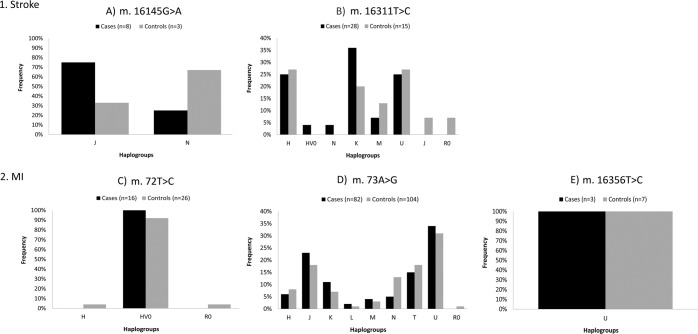
Distribution of fixed mutations between the mtDNA haplogroups. (**A**) *m.16145 G* > *A and* (**B**) *m.16311 T* > *C* for stroke group (1) and (**C**) *m.72 T* > *C*, (**D**) *m.73 A* > *G and* (**E**) *m.16356 T* > *C for MI group (2)*.

Haplogroup assignment was previously performed by Umbria *et al*.^24^. In Fig. 2 are shown the distribution of m.16145 G > A and m.16311 T > C in stroke group and m.72 T > C, m.73 A > G and m.16356 T > C in MI group taking into account the different mtDNA haplogroups.

The search of the mentioned positions in the updated mtDNA phylogeny – mit. Tree build 17^25^ shows that with the exception of m.72 T > C and m.73 A > G, which can be observed in several European haplogroups such as HV and H, the remaining positions are found in different branches of the phylogeny representative of haplogroups L1, L2, L3* and M1 characteristic of Africa; M5, M6, M31 and other M subgroups characteristic of South Asia; A, C, D, G, N9 and F characteristic of East Asia, even M29, Q, P and S characteristic of the Australian continent. In the present study (Fig. 2), positions m.16145 G > A, m.16311 T > C and m.73 A > G present a generalised distribution and can be observed in individuals belonging to haplogroups L, M, R0, H, HV, J, T, U, K and N. Therefore, the analysis of distribution of these mutations showed that they act as possible haplogroup-independent risk factors. By contrast m.72 T > C and m.16356 T > C have a high association only with the haplogroups HV0 and U, respectively. However, in this study there are individuals belonging to haplogrup HV0 that do not have the m.72 T > C. In the same line, note that all the individuals identified in this study with m.16356 T > C belongs to haplogroup U. Even though m.16356 T > C is found in different U subgroups (U2e3, U3a1c, U4 and U5b1). Hence, the analysis of the distribution of these mutations demonstrated that they were not associated with any particular mtDNA haplogroup. Therefore, these positions also could act as an independent genetic factor.

## Discussion

In western countries, where the burden of CVD is growing due to effect of CV risk factors, several studies have already shown the strongly relation of the genetic factors. However, little is known about the role of mtDNA CR mutations in development of stroke and MI^7–9,11,13–15,17^.

An association of several mtDNA alterations (fixed and in heteroplasmy) in the two diseases have been detected in the present study. As regards fixed mtDNA mutations, the set of mutations in stroke and MI cases was compared to controls and significant differences were found in the two diseases, reporting the m.16145 G > A and m.16311 T > C as a potential genetic risk factors for stroke, and m.72 T > C, m.73 A > G and m.16356 T > C as possible beneficial genetic factors for MI. It has been previously described that the CR mutations can be associated across multiple diseases, and that the same variant could had opposite effect (increase or decrease the risk) for two different diseases^9^. This finding would support our original hypothesis about the consequences that can affect the mtDNA mutations in the CR depending on the disease.

The CR variants do not act directly on the ETC affecting mitochondria bioenergetics or ROS generation; they may impact mtDNA transcription^18,19^ because contains the main regulatory sequences for replication initiation and transcription^3^.

Transitions 16145 G > A and 16311 T > C seem to have a pathogenic role in stroke. The analysis of distribution of these mutations clearly showed that they are located in many different haplogroups and consequently these mutations act as haplogroup-independent risk factors. The m.16145 G > A is located between MT-TAS sequence (nt. 16157–16172) and MT-TAS2 sequence (nt. 16081–16138). According to the classic strand-asynchronous mechanism, recent studies demonstrated that the 5'end of the D-loop is capable of forming secondary structures^26^, which act as a recognition site to molecules involved in the premature arrest of H strand elongation^27^. The biological importance of this region was confirmed by Brandon *et al*.^28^ who also observed multiple tumor specific mutations in the pre-TAS region. These observations suggest that mutations arising near to this conserved motive might be responsible of the alterations in mtDNA replication and transcription. In the same line, m.16311 T > C has been found to be significantly associated with certain types of cancer^29–31^. This mutation was previously described by Chen *et al*.^29^ in patients with prostate cancer and also has been reported in colorectal cancer^30^ and more recently in acute myeloid leukemia^31^. This mutation is located between the control elements Mt5 sequence (nt. 16194–16208) and the Mt3l sequence (nt. 16499–16506). In this case, our results showed that m.16311 T > C may implies a reduction in the stability of secondary structure of this region, which would affect in the binding grade to mtDNA transcription factors, ultimately affecting on the intensity of transcription regulation^32^. In both cases, these findings strongly suggest that mtDNA CR dysfunction may cause a decrease on the mtDNA copy number, which could affect the efficiency of ETC, lowering the ATP:ADP ratio and increasing ROS production^18,19^, contributing in stroke development.

Concerning MI, our results showed that m.72 T > C, m.73 A > G and m.16356 T > C act as a beneficial factor for MI. Although a high percentage of individuals with these mutations belonged to the haplogroups HV0, H or U, which have been shown to may have higher oxidative damage^9,24,33,34^, the distribution of mutations in positions 72, 73 and 16356 in our samples was independent of these haplogroups. Since the role of the mitochondrial genome in CVD susceptibility remains uncertain, it is difficult to explain how these mutations can decrease or counteract the progression of MI. Although m.72 T > C, m.73 A > G and m.16356 T > C have been previously related to certain types of cancer^35^, many studies consider that they are recurrent variants common in humans^36^.

Even though the most deleterious mutations are removed by natural selection, a wide range of milder bioenergetic alterations are introduced in certain populations^37^. Some of these variants as m.72 T > C, m.73 A > G and m.16356 T > C could be advantageous and seen as way to facilitate survival in specific environments. In contrary, other mutations as m.16145 G > A and m.16311 T > C escape of intraovarian selection and could cause significant mitochondrial defects and stroke development. Much of the progress in linkage disequilibrium mapping of complex diseases has been made using the major assumptions of the CDCV hypothesis, that is, that common alleles cause common diseases. After found positive associations with common alleles (e.g., those found by Umbria *et al*.^24^), it was necessary to replicate the results and then look for rarer variants, with potentially greater penetrance. However, all the mutations analysed in the present study had a minor allele variant >5% (common variant) or between 1–5% (low-frequency). Although common variants are often associated with OR between 1.2 and 1.5^38^, our results showed higher effect size for pathogenic variants, with OR values of 2.4–4.4, and similarly, higher protective effect size of variants found at higher frequency in controls (ORs < 0.5) relative to cases.

Our findings also showed a significant increase of point heteroplasmy in MI controls in comparison to cases. This result is contrary to expectations, because the presence of heteroplasmy has been commonly associated with aging and degenerative diseases, due to a decline in mitochondrial function in both these processes^39^. Our registered heteroplasmic positions (16399, 16129, 16093, 16092, 73, 146, 150, 152, and 204) were located in hotspots positions of the hypervariable segments. Recent evidences demonstrate that an important fraction of mutations detected in heteroplasmy are germinal or originated in very early stages of the development^40^. Moreover, it is probable that germinal heteroplasmy has a beneficial or risk effect, and our results revealed that most of point heteroplasmy were overrepresented in MI controls individuals. This fact, is no surprise because some heteroplasmic positions detected, as m.73 G > A, have been linked as possible beneficial genetic factors for MI and also it has been suggested that other heteroplasmic positions, such as 146 T > C, 150 C > T or 152 T > C may increase longevity^41^.

Many studies have shown that heteroplasmic variants without apparent genetic or cellular functional consequences are observed in apparently healthy individuals^42–45^. In the present study, the frequency of MI conrols with point heteroplasmy in the CR (5.2%; 95% CI [0.045–0.972]) are slightly higher than those reported by Santos *el at*.^44^ (3.81%; 95% CI [0.166–0.737]), but less than described by Ramos *et al*.^45^ (7.9%; 95% CI [0.041–0.149]), demonstrating that heteroplasmy occurs with appreciable frequency in the general population^42^. This idea is even more reinforced in front of the present data, since the higher representation of point heteroplasmies detected were located in non-stable positions and by the fact that no significant differences were found between frequencies of point heteroplasmy in stroke cases and controls.

It should be noted that this study has some limitations and further functional analysis may be required in independent large samples to clarify the contribution of mitochondrial control region variants to stroke and MI development. At the moment there are no experimental models that prove that alterations in these variants really have consequences. In fact, there are mtDNA regulatory sequences that have been assigned to control region although they remain practically unproved. In addition, it must also be taken into account that measurement of mitochondrial genetic variation in blood may differ from that in cardiovascular tissues. Another important point is that, although the population specificity of mtDNA exists, it has been observed that positions reported in this work are worldwide distributed (m.16145 G > A, m.16311 T > C and m.16356 T > C) or at least, in several Eurasian haplogroups such as HV and H (m.72 T > C and m.73 A > G) (http://www.phylotree.org/). For this reason, it would be interesting to explore if our results are extrapolable to other populations, since in a different genetic context, these positions might not present the same effects.

## Conclusions

In conclusion, the statistically significant differences in the frequencies of variants in the mitochondrial control region sequence between stroke and MI cases and controls at five positions provide new evidence and better understanding of the cellular mechanism by which mtDNA variants contribute to CVD, and endorse the importance of including this regulatory region of the mtDNA in genetic association studies.

## Material and Methods

### Patients and samples

In this study, data from 730 subjects (154 individuals with stroke history, 211 individuals with MI history and their corresponding control individuals -matched for age (categories ≤ 44, [45–49], [50–54], [55–59], [60–64], ≥ 65 years), sex and geographic origin (North, Central and South regions of Castile and Leon), were used. Samples come from the Cardiovascular Disease Risk Study of Castile and Leon^46^ in a cross-sectional, observational and descriptive study performed in Castile and Leon (centre-north region of Spain), whose design and analysis have already been described by Umbria *et al*.^24^.

For each individual, we also obtained information about history of hypertension (≥140/90 mmHg), history of diabetes, history of hypercholesterolemia (>200 mg/dl), cigarette consumption (smokers, former smokers and non-smokers), presence of overweight or obesity (body mass index ≥ 25 kg/m2), presence of high abdominal perimeter in risk range (risk: ≥80 cm for women and ≥94 cm for men) and presence of high levels of triglycerides (≥170 mg/dl). The study was approved by the ethical committee from Universitat Autònoma de Barcelona and appropriate informed consent was obtained from all the individuals.

### MtDNA sequence analysis and heteroplasmy authentication

The mtDNA sequences used in the present study were previously obtained by Umbria *et al*.^24^ although, they were strictly used to classify samples into mtDNA haplogroups. In brief, the control region of the mtDNA was amplified and sequenced between positions 15907 and 580 using primers and conditions described by Santos *et al*.^44^. Moreover, coding region phylogenetic mtDNA informative polymorphisms 7028, 11719, 12308, 12705, 13708 and 14766 were further analyzed as previously detailed by Santos *et al*.^47^, to improve haplogroup classification.

In the present study, sequences were reassessed and analysed at the nucleotide level to identify not only fixed mutations but also mutations in heteroplasmy. The alignment in relation to the to the revised Cambridge Reference Sequence (rCRS)^48^ and the heteroplasmy detection were performed using the SeqScape 2.5 software (Applied Byosistems, Foster City, USA) considering a value of 5% in the Mixed Base Identification option. Only sequences with satisfactory peak intensity and without background/noise were considered. In this context, some samples were amplified and sequenced several times (using the same methodology described in Umbria *et al*.^24^) to obtain accurate sequences to heteroplasmy detection. Moreover, additional analyses were performed in order to authenticate heteroplasmies.

The authentication of mtDNA heteroplasmy was performed following a similar strategy to that used by Santos *et al*.^44,49^.

1. PCR amplification and sequencing of the control region of the mtDNA.
2. To authenticate the results for samples presenting heteroplasmy in step 1, a second PCR amplification and sequencing were performed.
3. In addition, to exclude a possible contamination of the samples, an analysis of Short Tandem Repeat (STR) DNA profiling was carried out employing AmpFlSTR® Identifiler® PCR Amplification Kit (Applied Biosystems, Foster City, USA) following the manufacturer’s protocol.

Thus, point heteroplasmic positions were accepted if they appeared in all the validation steps and no evidence of sample contamination was detected.

Levels of heteroplasmy were determined using the height of peaks in the electropherograms^44^. To calculate the average heteroplasmic levels, the results obtained for at least two sequence reads of each heteroplasmic position were used.

### Data analysis

#### Statistical analyses

To compare differences in the CR profile between cases and controls in both stroke and MI, all fixed and heteroplasmic mtDNA mutations were compiled into a matrix considering cases and controls analysed for each disease.

All fixed mtDNA mutations detected in cases and controls (present in a minimum of 10 individuals) were tested together by using a conditional logistic regression analysis (forward stepwise model), adjusting the association analysis for the potential confounding effect of CV risk factors previously detected by Umbria *et al*.^24^. The authors used McNemar’s test or marginal homogeneity test to compare the frequency of sociodemographic, biochemical and clinical characteristics above mentioned between stroke and MI cases and controls (Table 5). Hypercholesterolemia was considered a CV risk factor with a potential confounding effect for stroke, while both hypertension and hypercholesterolemia were considered for MI samples. Therefore, Odds Ratios (ORs) and their 95% Confidence Intervals (CIs) were calculated adjusting for the effect of these risk factors in each disease. To compare the presence or absence of point and length heteroplasmy, a logistic regression analysis was used to correct for the effect of CV risk factors above mentioned^24^.

**Table 5:** Sociodemographic, biochemical and clinical characteristics of stroke and myocardial infarction cases and controls.

	Stroke Controls	Patients with stroke	p-value^a^	MI Controls	Patients with MI	p-value^a^
	n = 154 (%)	n = 154 (%)		n = 211 (%)	n = 211 (%)	
Age (mean ± SD, years)	71.4 ± 8.6	73.9 ± 10.5	Matched	68.9 ± 11.1	71.2 ± 12.1	Matched
Male/female	91 (59.1)/ 63 (40.9)		Matched	128 (60.7)/ 83 (39.3)		Matched
Geographic origin (N/C/S)	40 (26)/ 75 (48.7)/ 39 (25.3)		Matched	54 (25.6)/88 (41.7)/69 (32.7)		Matched
Ever smoking (n/N, %)	18/154 (11.7)	16/154 (10.4)	0.448	33/211 (15.6)	24/211 (11.4)	0.566
Hypertension (≥ 140/90 mmHg)	120 (77.9)	134 (87)	0.055	146 (69.2)	191 (90.5)	< 0.001
Diabetes	22 (14.3)	32 (20.8)	0.144	40 (19)	54 (25.6)	0.099
Hypercholesterolemia ( > 200 mg/dl)	64 (41.6)	92 (59.7)	0.002	100 (47.4)	151 (71.6)	< 0.001
Overweight or obesity (≥ 25 kg/m2)	118 (76.6)	103 (66.9)	0.092	162 (76.8)	158 (74.9)	0.734
High abdominal perimeter (> 80 or > 94 cm)	120 (77.9)	108 (70.1)	0.169	163 (77.3)	159 (75.4)	0.731
Triglycerides (≥ 170 mg/dl)	14 (9.1)	15 (9.7)	1.000	28 (13.3)	27 (12.8)	1.000

MI: Myocardial infarction, SD: Standard deviation, N: North, C: Central, S: South

P-value of a paired McNemar test for dichotomous variables samples and Marginal Homogeneity test when a category of the samples is more than two.

^a^p-value of McNemar or marginal homogeneity test used to compare stroke and MI cases and controls.

Finally, mtDNA mutations were revised to infer if they were located in haplogroup-defining positions (previously examined in Umbria *et al*.^24^), or acts as an independent genetic factor.

Statistical analyses were performed using IBM SPSS ver. 22.0 (SPSS Inc.). All differences were considered significant at p < 0.05.

#### Hits in the phylogeny, population database and Conservation Index (CI)

The stability of fixed mtDNA mutations and point heteroplasmic position were analysed as previously detailed by Ramos *et al*.^45^. The number of hits in the phylogeny for each position was compiled from the updated mtDNA phylogeny – mit. Tree build 17^25^ – and from Soares *et al*.^50^. From these data, it has been possible to calculate the probability of mutation as the ratio between the observed and the total number of hits. An mtDNA position was considered a hotspot if the mutation probability was ten times higher than the expected mean value. In order to calculate the frequency of each variant for a particular nucleotide position, a database of 3880 mtDNA complete sequences was used. Sequences were aligned using Clustal W and formatted for further frequency analyses using the SPSS software. The nucleotide conservation index (NCI) was estimated only across reference sequences of different primate species (for the list of species and accession numbers see Supplemental Table S2). Sequences were analyzed using the same method previously mentioned^45^.

#### Structure prediction

Secondary structures were performed to understand the structural impact of the different variants found. Secondary structures for each position were generated from sequences (A-M) identified by Pereira *et al*.^26^. All sequences were submitted to the RNAfold web server (http://rna.tbi.univie.ac.at/cgi-bin/RNAWebSuite/RNAfold.cgi) using default parameters for DNA secondary structures calculations. The minimum free energy prediction and base pair probabilities were used to estimate the implication in the molecule.

## Supplementary information

Supplementary Table S1.

Supplementary Table S2.

## Data Availability

The data that supports the findings of this study are within the paper and its Supplementary Material File.
